# Effect of Glass Fiber Post Surface Treatment on Bond Strength of a Self-Adhesive Resin Cement: An “In Vitro” Study

**DOI:** 10.1155/2021/8856657

**Published:** 2021-08-24

**Authors:** Lairds Rodrigues dos Santos, Darlon Martins Lima, Edilausson Moreno Carvalho, Vandilson Pinheiro Rodrigues, Claudia Maria Coelho Alves

**Affiliations:** ^1^Graduate Program in Dentistry, Federal University of Maranhão, São Luis, Maranhão, Brazil; ^2^Department of Dentistry I, Federal University of Maranhão (UFMA), São Luís, Brazil; ^3^Department of Morphology, Federal University of Maranhão (UFMA), São Luís, Brazil; ^4^Department of Dentistry II, Federal University of Maranhão (UFMA), São Luís, Brazil

## Abstract

**Objective:**

This study evaluated the influence of different mechanical and chemical surface treatments alone and combined with silane on the bond strength (BS) of glass fiber posts (GFPs) using self-adhesive resin cement.

**Methods:**

Eighty-four single-rooted bovine teeth (six groups, *n* = 14) were submitted to BS analysis after GFP cementation. The treatments applied in the studied groups were no surface treatment (control), silane (S), 24% hydrogen peroxide (PER), 24% hydrogen peroxide and silane (PER + SIL), blasting with 50 *μ*m aluminum oxide particles (BLAST), and blasting with 50 *μ*m aluminum oxide particles and silane (BLAST + SIL).

**Results:**

BS differed significantly among groups (*p* < 0.001). It was higher in the SIL (10.5 ± 3.5 MPa), BLAST + SIL (11.5 ± 3.2 MPa), and PER + SIL (11.6 ± 4.6 MPa) groups than in the control (6.5 ± 2.9 MPa), BLAST (8.6 ± 4.0 MPa), and PER (7.1 ± 2.8 MPa) groups, with no significant difference among groups receiving silanization. Cement post adhesive failure was more common in the SIL, BLAST, and PER + SIL groups, and cement-dentin adhesive failure was more common in the control, BLAST + SIL, and PER groups.

**Conclusion:**

These results show that silane application alone increases BS.

## 1. Introduction

The use of glass fiber posts (GFPs) has increased in daily practice [1] and is recommended to promote additional retention of core buildups and direct restorations [2]. However, retention of the post in the root canal depends on the bond strength (BS) at different parts of the dentin-cement interface, as loss of retention of this type of post is more common than root fracture [2–4].

GFP restorations have more prolonged survival [5] and post adhesion to dentin and resin cement plays a vital role in restoration longevity [6]. Chemical and mechanical treatments of GFP surfaces seem to influence the BS between the post and resin materials [7, 8]. Thus, the effective BS between a fiber-reinforced system and resin cement is crucial [9]. Several chemical and mechanical surface treatment protocols have been developed to improve the surface energy of fiber-reinforced posts [4, 7, 9, 10].

The BS between a post and resin cement can be improved chemically by silanizing the post surface, and no other treatment is necessary before silane application [11, 12]. However, research has shown that silane alone does not increase the BS of GFPs to resin cement, as the surface roughness produced by other surface treatments performed before silane application is essential for improving chemical or mechanical retention at the post-resin cement interface [13–15].

Researchers disagree about the actual benefit of silanization in improving GFP retention, and other surface treatment alternatives have been investigated [11, 14]. The roughness produced on the post surface with mechanical (*e.g*., blasting) [14, 16] or chemical (*e.g*., application of hydrogen peroxide) methods [16, 17] has been shown to improve the retention of GFPs fixed with resin cement by removing the matrix layer of epoxy resin and increasing the area of contact with the fibers that will be silanized [18, 19].

Nevertheless, despite laboratory studies [10, 20–22] on GFP surface treatments, there is no consensus on the most effective treatment to achieve optimal adhesion. Thus, this *in vitro* study aimed to evaluate the influence of different chemical and mechanical surface treatments, alone and in combination with silane, on the BS of GFPs fixed with self-adhesive resin cement.

The null hypothesis was that the application of silane alone and in combination with other chemical and mechanical treatments would not improve the BS at the post-resin cement interface.

## 2. Methods and Materials

### 2.1. Preparation of Teeth

Eighty-four uniradicular bovine incisors were used in this study. The root canals were treated endodontically with K-files (Dentsply Maillefer, Petrópolis, RJ, Brazil) to the apical limit using the crown-down technique. The root canal cervical diameters were measured in the mesiodistal and linguovestibular directions with a pachymeter. An average of these measurements was calculated, and the root canals with a cervical diameter equal to 2 mm (±1.0) were selected. Irrigation was performed with 1 ml saline solution (0.9% NaCl) at the time of each file exchange during mechanical preparation. The canal space was filled using the conventional technique. The roots were stored in distilled water at 37°C for one week for total cement prey.

**Figure 1:**
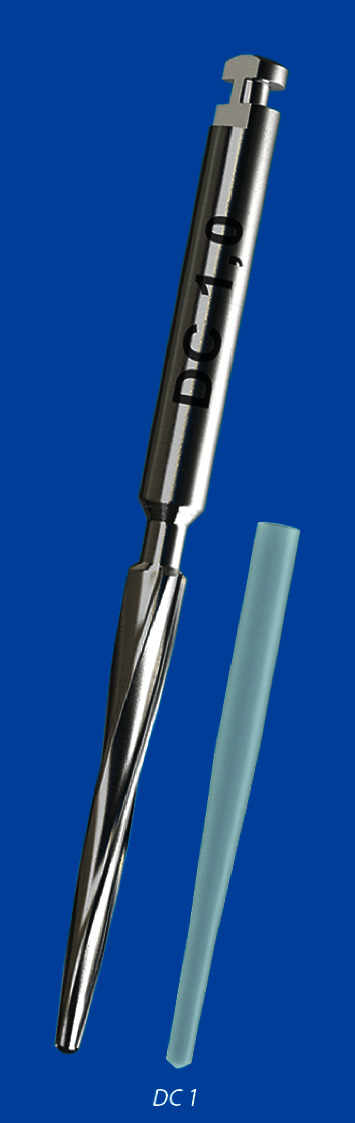
White post DC #1 and corresponding drill. Source: photo courtesy of the FGM dental group product image bank.

The root canals corresponded to the diameter of drill # 1 of the White Post pin kit, graduated to the depth of 12 mm. The pins used for cementation were White Post DC # 1 according Figure 1 (FGM, Produtos Odontológicos Ltda., Joinville, SC, Brazil).

The roots were stored in distilled water and placed in an oven at 37°C for seven days to allow for the complete cement setting. Then, the pins were released with the maintenance of an apical sealing of 5 mm filling material at the apex to minimize internal dentin removal when the post was placed. The radicular canals were washed with distilled water and dried with absorbent paper cones to remove dentin debris.

### 2.2. Experimental Groups and Surface Treatments

The roots were divided into six experimental groups (*n* = 14 each), and the posts were submitted to different surface treatments: none (control), silane (SIL), 24% hydrogen peroxide (PER), 24% hydrogen peroxide and silane (PER + SIL), blasting with aluminum oxide (BLAST), and aluminum oxide blasting and silane (BLAST + SIL). The posts used in this study (White Post DC #1; FGM, Produtos Odontológicos Ltda.) had smooth surfaces and a length of 20 mm.

Before surface treatment, all posts were tested in the root canals, cleaned with 70% hydrated ethyl alcohol (Rioquímica Indústria Farmacêutica Ltda., São José do Rio Preto, SP, Brazil) for 1 min, and air dried. The post surfaces were then treated, and adhesive (Adper Single Bond 2; 3M ESPE, Sumaré, SP, Brazil) was applied according to the manufacturer's recommendations. The protocols are described in Table 1.

### 2.3. Post Cementation

The posts were cemented in the root canals after surface treatment and adhesive application. Cementation was performed in a single direction using Relyx U200 self-adhesive resin cement (3M ESPE, St. Paul, MN, USA), following the manufacturer's recommendations. The cement was inserted into the cavity with the aid of a syringe (Centrix Inc., DFL Indústria Química, Rio de Janeiro, Brazil), starting in the apical third of the canal, with constant movement toward the cervical third until the occurrence of cement extravasation, to avoid the presence of bubbles, which affect the adhesion process [15]. Excess cement was removed with an applicator tip. Photopolymerization was implemented with the tip of a photopolymerizer (Demilled; Kerr Corporation, Middleton, WI, USA) with an intensity of 800 mw/cm^2^ and vertical application of the light source to the post for 40 seconds (s). A composite resin layer (Z350XT; 3M ESPE, St. Paul, MN, USA) was placed on the cervical surface of each root and photopolymerized for 40 s. The portion of the post that remained outside the conduit was not cut. The roots were stored in distilled water and placed in an oven at 37°C to allow for a complete cement setting.

### 2.4. Specimen Sectioning and Push-Out Test

Following post cementation, roots were fixed on acrylic plates with sticky wax (Cerafix; Indústria e Comércio de Artigos Odontológicos Ltda., SP, Brazil). They were sectioned using a universal precision cutting machine (Iso Met 1000; Buehler, Lake Bluff, IL, USA) at low speed under irrigation with distilled water and a double-sided diamond disc (no. 7020, 022 mm; KgSorensen, Cotia, SP, Brazil). Sectioning was performed in the apical direction perpendicular to the root axis, producing two slices of each third of the root (cervical, middle, and apical) with thicknesses of approximately 1.4 mm (±0.1). The first cut was made at 1.4 mm (±0.1) on the most cervical portion of the root for standardization of measurement with a 0.01 mm precision digital caliper. The cervical and apical sides of each slice were photographed at 30x magnification (Kozo Optical and Electronical Instrumental, Nanjing, China) using an external light source (XZ-150 WA Cold Light Illuminator, Ted Pella).

The cervical and apical root canal diameters were measured on each slice using Image J software (version 1.46; National Institutes of Health, USA). The push-out test was conducted in a universal testing machine (3342; Instron, USA) with a speed of 0.5 mm/min.

### 2.5. Fracture Pattern Analysis

The specimens were evaluated under a stereomicroscope at 30x magnification (Kozo Optical and Electronical Instrumental, Nanjing, China) after mechanical testing. Failures were classified using four categories: failure of the adhesive between the resin cement and post (ACP), failure of the adhesive between the resin cement and root dentin (ACD), mixed failure (MIX), and cohesive failure in dentin (COE).

### 2.6. Scanning Electron Microscopy Analysis

GPFs were prepared in the same way as for the push-out test. The samples were fixed in stubs, and images of the morphological structure of the posts were obtained by Scanning Electron Microscopy (SEM) (Model TM3030, Hitachi High-Technologies, Tokyo, Japan) at 15 kV and 1000x magnification.

### 2.7. Statistical Analysis

Data were analyzed using SPSS software (version 17.0, IBM, Chicago, IL, USA). The normality of the distribution of numerical variables was assessed using the Shapiro–Wilk test. The analysis of variance, followed by Tukey's test, was used to compare BS between the study groups and root thirds. Fracture patterns were compared among study groups and root thirds using the Fisher exact test. The level of significance was set to 5% (*p* < 0.05).

## 3. Results

Table 2 shows the comparative analysis of bond strength (BS) between study groups and root thirds. There were statistically significant differences among groups at all the three root thirds and overall (*p* < 0.001).

The highest measurements of BS statistically were observed for SIL, BLAST + SIL, and PER + SIL at all three root thirds (*p* < 0.05). Silane application promoted a superior BS than nonsilanized pins. An increase in BS was identified when the silane group is observed alone or in association. Although the associated treatments show a tendency to increase BS values, no significant difference was observed between them. The control group, peroxide, and blasting obtained the lowest BS values and were statistically similar.

The highest BS values were found for silane, blasting, silane and peroxide, and silane groups, but there was no statistically significant difference between the groups.

ACP failure occurred more frequently in BLAST than the control group at the cervical root third (*p* < 0.05). ACP failure was more frequent in SIL than the control group at medium root third. Significant differences were also observed between cervical and apical root thirds for all experimental groups (*p* < 0.05). ACP failure was the most common failure type in the cervical third, whereas ACD failure was the most prevalent type in the apical third (Table 3).

**Figure 2:**
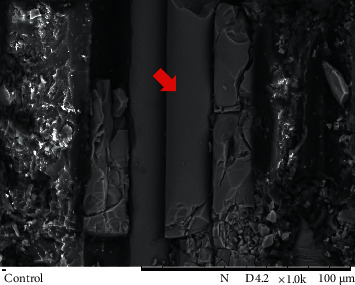
SEM image of a post surface from the control group (1000x magnification). Areas of intact fibers are visible, but discontinuity is present (arrow).

**Figure 3:**
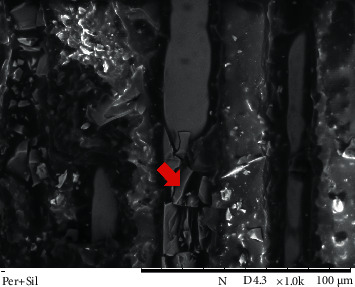
SEM image of a post surface from the peroxide and silanization group (1000x magnification). Areas with an irregular morphological aspect, indicating surface roughness, which facilitated wetting by silane and the flow of resinous cement, are visible (arrow).

**Figure 4:**
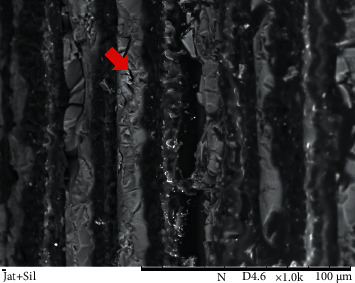
SEM image of a post surface from the blasting and silanization group (1000x magnification). Areas with an irregular morphological aspect, indicating surface roughness, which facilitated wetting by silane and the flow of resinous cement, are visible (arrow).

Representatives SEM images from the post surface treatment protocols used in study groups are shown in Figures 2–4.

## 4. Discussion

The ability of chemical and mechanical treatments to improve the BS of GFPs to resin cement, alone and combined with silane application, was investigated in this study. The bond strength was affected by surface treatment, and silane application improved BS compared to values obtained for nonsilanized posts. Thus, the null hypothesis was rejected.

BS was lower in the groups that did not receive silane treatment in this study. In the control group, a failure probably occurred between the smooth surface and the low surface energy of the untreated post and resin cement [23] as no bonding is expected to occur between the methacrylate-based resin cement and the GFP's epoxy resin matrix [24]. In the PER and BLAST groups, the roughening of the post surfaces, which exposed the glass fibers, did not increase BS.

Several researchers have proposed silane application to post surfaces to increase the BS between GFPs and resin cement [11, 12, 21, 22]. Silanes are bifunctional molecules with one end capable of reacting with inorganic fiberglass and another one capable of copolymerizing with organic resin. Silanization increases surface wettability as intimate contact with the materials is established, creating strong bonds with hydroxyl groups with inorganic substrates of the fiberglass posts [11, 12].

The roughness produced by the combination of treatments exposes the glass fibers, enabling better chemical bonding with silane and micromechanical bonding with resin cement [2, 18], which would probably increase BS. In this study, the use of silane alone and in combination with other chemical and mechanical treatments increased BS, clearly confirming the benefits of silanization.

Although the surface treatments associated with silane tend to increase BS values concerning silane alone, this trend was not significant. This may be because there may have been a compromise in the polymerization of the resin in the PER group due to the formation of free radicals and the layer rich in oxygen by-products [22]. In the BLAST group, this procedure may have caused some damage to the glass fibers, which compromises pin integrity and treatment longevity [25], factors that prevented a more significant increase in BS compared to silane alone. Our findings agree with previous studies [21, 22, 26, 27], which have determined that treatment with silane alone is enough to improve BS between cementing agents and GFPs. The first explanation for this finding is that solid covalent bonds are created between silane and resin composites and silica fibers [2]. The silanization also increases the surface energy of GFPs due to increased surface wettability [28]. In this study, silane was used in combination with an adhesive layer, and both materials can infiltrate the pin's surface and reinforce the bond with the resin cement, improving the UK. This was an advantage shown in this work. According to Rechia et al. [29], when silane is used in isolation, it can form a nonhomogeneous layer on the surface of the pins. The second explanation is that the brand of GFP may influence the BS, as some posts have more exposed fibers on their surfaces compared with others, which are more superficially protected by the epoxy resin matrix [2, 22, 30, 31].

The high BS values observed in the SIL group in this study, which received no treatment that would increase the post surface's roughness, can be explained by the presence of naturally exposed fibers in some areas of the GFPs, which provide chemically reactive sites for silane bonding [2, 6, 22, 32]. The surfaces of the GFPs used in this study have large amounts of exposed fibers; the manufacturer reports 80% and 20% of glass fiber weight and epoxy resin, respectively, leading to higher BS in the SIL group. Thus, we can consider that one of the merits of this study was to show that the manufacturing process can determine the structural characteristics and mechanical properties of PFV. Novais et al. [33] state that there is a linear correlation between strength and fiber/matrix ratio and the flexural modulus and the number of fibers. Higher BS values have been reported to treat GFPs whose surfaces are covered with more epoxy resin matrix [10, 34]. The authors of these studies stated that the type of post used had few functional groups to react with the silane coupling agent and, thus, that silanization had a more negligible influence on BS, whereas surface roughening led to greater exposure of the glass fibers and absorption of silane, increasing BS. However, even with the removal of the superficial layer of epoxy resin matrix by the surface treatments used in this study, the chemical alteration of the post surface caused by silanization had a more significant effect on BS than the increased surface roughness.

Uniformity at the interface between the matrix and the fiber is essential for pin integrity. A discontinuous morphological post structure was noted, which may have resulted in low BS values. Exposed fibers can reveal damage to the outer surface/manufacturing flaws [25], affecting the pin's mechanical strength. Posts treated with hydrogen peroxide and aluminum oxide blasting showed surface roughness with partial removal of the epoxy resin matrix compared to the control group (Figure 2). However, the presence of microporosities on the post surface was not enough to significantly improve BS.

The SEM images of post surfaces subjected to mechanical treatment followed by silanization (Figures 3 and 4) show that the spaces created not only exposed the fibers but also facilitated the reaction of the silane with the fibers and improved resin cement flow, leading to greater bonding and increased BS between the post and composites.

We observed greater BS in the cervical region of the root canal than in other regions. These results agree with the work of Alshahrani et al. [17], in which the RelyX U200 cement showed higher BS values in the cervical third, regardless of the type of surface treatment of the post. The success of adhesive cementation inside the root canal, mainly at the apical level, can be compromised by several factors. The nonuniformity of dentin hybridization and resin tags more frequently in the cervical region than in the apical region, where the tubular density is lower [9, 35, 36], favor the cervical region regarding BS [8, 19]. Other influencing factors include greater access to the cervical portion of the canal for cleaning, light transmission into the canal walls, and humidity control.

All types of failure were observed in this study, with differences according to the surface treatment. A large amount of ACD failures in the apical region can be explained by the possible presence of a residual smear layer, gutta-percha, and endodontic cement, which prevent adequate contact between the dentin and resin cement [19]. The cement layer may also not have been homogeneous at this interface, reducing the adhesion surface area. The large number of ACP failures found in the cervical root thirds reflects greater cement homogeneity at this interface, residue free.

The great diversity of surface treatment protocols, adhesives, and resin cement used in *in vitro* studies [13, 30, 35] hinders comparison with the results of this research. However, few studies [9, 14, 26] have used well-established criteria, such as the use of teeth free of cracks, fractures, curvature, open apices, caries, and restorations; use of materials according to the manufacturer's instructions; use of similar-dimension teeth (determined radiographically); and endodontic treatment performed by the same operator. Observing the effects of chemical and mechanical treatments alone and in combination with silane and in comparison with the use of silane alone and with a control group, this study showed the importance of silanization and the need for combined treatments to increase BS.

A differential of this study was that the application time and the materials used for the surface treatments could be carried out during clinical care. Proper surface treatment is essential for successful restoration [8]. In this study, the surface treatment before silanization to improve adhesion of GFPs to resin cement should be interpreted with caution since the treatments used in combination with silane were compared to using silane alone. The composition of GFPs used for cementation should also be investigated, considering that this study showed that an essential factor for the resistance of PFV might be the amount of matrix and the type of treatment used to promote the chemical bond between the fiber and the resin.

On the other hand, this work had some limitations. There were no other commercial brands of PFV used to compare the UK with the different surface treatments evaluated. Different brands of PFV have different chemical compositions and structures, including epoxy resin and the arrangement of the fiberglass within the pin. Thus, controversies in the conclusions with previous studies are probably related to differences in the composition of the pins, time of application, and concentrations of surface treatments.

The results of this study indicate that silanization of GFPs improves BS and is a step that cannot be ignored. However, the need to perform more complex treatments that require more dentist office time depends on post composition. Therefore, adopting the manufacturer's recommendations avoids neglecting steps, and adhesion can be compromised when the protocol is not carried out selectively. The amount of epoxy resin matrix naturally found in GFPs influences the need to associate chemical or mechanical treatments with silane. Thus, the use of silane alone for the post used in this study is a protocol that provides satisfactory results for adhesion at the cement-post interface.

## 5. Conclusions

This *in vitro* study revealed a significant improvement in BS in the posts that received silanization. For the GPFs used in this study, using other chemical and mechanical treatments in combination with silane is not an indispensable and essential condition for increasing BS.

## Figures and Tables

**Table 1:** Glass fiber post surface treatment protocols.

Groups	GFP surface treatment
Control (C)	No surface treatment

Silane (SIL)	Silane layer was applied to the post surface (silane/Dentsply) for 1 minute with the aid of a microbrush (GN/Injecta) following the manufacturer's recommendations. Later, the adhesive system was applied

Peroxide (PER)	The post surface was immersed in 24% H_2_O_2_ solution for 1 minute. Then, they were rinsed with distilled water and dried with air blasts. The adhesive system was then applied

Hydrogen peroxide and silane (PER + SIL)	The post surface was immersed in 24% H_2_O_2_ for 1 minute. Then, they were rinsed with distilled water and dried with air blasts. Silane application was made, and then, the adhesive system was applied

Aluminum oxide blasting (BLAST)	The posts were blasted using a blasting machine (Microjato/Gold Line) with aluminum oxide particles of 50 *μ*m (Bio-art) ejected at 1 cm perpendicular to the surface of the post to make superficial microretentions for 5 seconds. The pressure exerted during blasting was 0.3 MPa. The posts were washed with distilled water and dried with air blasts, and then, they received the application of the adhesive system

Aluminum and silane blasting (BLAST + SIL)	Blasting was performed on the post as described above, and then, they were conditioned by the silane and adhesive system (same technique)

**Table 2:** Mean and standard deviation (±sd) of bond strength (in MPa) for the study groups and evaluated thirds.

Root region	Control Group	Experimental groups					*p* ^*∗*^
		SIL	BLAST	BLAST + SIL	PER	PER + SIL	
	Mean (±sd)	Mean (±sd)	Mean (±sd)	Mean (±sd)	Mean (±sd)	Mean (±sd)	
Cervical	9.5 (2.4)^Aa^	12.6 (3.3)^BCa^	10.3 (3.5)^ACa^	14.2 (3.2)^Ba^	9.4 (2.4)^Aa^	13.8 (4.3)^Ba^	<0.001
Medium	6.0 (2.0)^Ab^	10.2 (3.2)^Bb^	9.8 (3.0)^Ba^	10.5 (2.0)^Bb^	6.7 (2.2)^Ab^	11.8 (4.9)^Bab^	<0.001
Apical	4.7 (2.1)^Ab^	8.8 (3.0)^Bb^	5.6 (3.7)^Ab^	9.8 (2.3)^Bb^	5.2 (2.0)^Ac^	9.1 (3.9)^Bb^	<0.001
Total	6.5 (2.9)^A^	10.5 (3.5)^B^	8.6 (4.0)^C^	11.5 (3.2)^B^	7.1 (2.8)^AC^	11.6 (4.6)^B^	<0.001

*p* was calculated using the ANOVA test followed by Tukey's test. Different upper-case letters represent statistically significant differences (*p* < 0.05) between treatment types in the same third root. Different lowercase letters represent statistically significant differences (*p* < 0.05) between the third roots with the same treatment group.

**Table 3:** Percentage distribution of the fracture pattern by the group and by evaluated third root.

Variables	Percentage of fracture pattern study group				*p*
	ACP (%)	ACD (%)	MIX (%)	COE (%)	
Group					0.014^∗^
Control group	43.9	46.4	3.6	7.1	
SIL group	57.1	36.9	0	6.0	
BLAST group	64.3	35.7	0	0	
BLAST + SIL group	44.0	51.2	0	4.8	
PER group	45.2	51.2	2.4	1.2	
PER + SIL group	50.0	46.4	0	3.6	

Third root					<0.001^∗^
Cervical	71.4	19.0	1.9	7.7	
Medium	53.0	44.6	0.6	1.8	
Apical	27.4	70.2	0.6	1.8	

^*∗*^Statistically significant percentage differences (*p* < 0.05) through the chi-squared test.

## Data Availability

No data were used to support this study.
